# Formulation of Emulsion Gels Stabilized by Soy Protein Isolate and Gellan Gum Conjugates for 3D Printing and β-Carotene Encapsulation Applications

**DOI:** 10.4014/jmb.2607.07035

**Published:** 2026-10-08

**Authors:** Eui-Jung Han, Sangeun Park, Jiyoung Park, Jae-Hee Lee, Yourim Oh, Jin-Kyu Rhee

**Affiliations:** 1 Department of Food Science and Biotechnology, Ewha Womans University, Seoul 03760, Republic of Korea; 2 SuFAB Inc., 123 Digital-ro 26-gil, Guro District, Seoul 04790, Republic of Korea

**Keywords:** Maillard conjugates, Emulsion gels, Soy protein isolate, Gellan gum, Encapsulation, 3D printing

## Abstract

Soy protein isolate–gellan gum (SPI–GG) conjugates were prepared at different mass ratios to investigate how protein–polysaccharide composition affects the physicochemical, emulsion, rheological, and functional properties of CEGs. Increasing the GG proportion enhanced the degree of glycation and Maillard browning while producing opposite trends in solubility and surface hydrophobicity. All SPI–GG CEGs exhibited smaller droplets, more negative ζ-potential values, and higher emulsifying activity and stability than the SPI control. Among the formulations, S1G1 showed the smallest and most uniformly distributed droplets, the highest emulsifying performance, and the greatest β-carotene encapsulation efficiency (90.94 ± 0.55%) and bioaccessibility (68%). In contrast, rheological and printing performance showed a different composition-dependent response. All CEGs exhibited shear-thinning behavior, solid-like viscoelasticity, and thixotropic recovery above 70%. CEG-S1G1.5 exhibited a combination of relatively high G*, critical strain, and yield stress, together with favorable dimensional retention after printing. These findings demonstrate that the SPI–GG ratio differentially affects emulsion characteristics and bulk rheological properties. Overall, the SPI–GG composition associated with favorable functional performance differed according to the intended application, providing a basis for composition-driven design of CEGs for 3D printing and lipophilic bioactive delivery.

## Introduction

Emulsion gels are structured multiphase systems in which oil droplets are immobilized within a viscoelastic or gel-like matrix [1]. In food applications, they can convert liquid oils into semi-solid structures while providing physical stability, adjustable rheological properties, and the capacity to incorporate lipophilic compounds [2]. The functional performance of emulsion gels depends on both emulsion-level characteristics, including droplet organization and physical stability, and the rheological and mechanical properties of the surrounding matrix [3]. These two property domains can play different roles depending on the intended food application. Therefore, simultaneous consideration of emulsion characteristics and bulk rheological behavior is important for designing emulsion gels for different food applications.

Soy protein isolate (SPI) is widely used as a plant-based food emulsifier because its amphiphilic structure enables adsorption at the oil–water interface [4]. However, SPI alone may not provide sufficient or independently adjustable control over both droplet stabilization and the bulk structural properties required under different processing and storage conditions [5]. The incorporation of polysaccharides is commonly used to modify protein-based emulsion systems because polysaccharides can increase aqueous-phase viscosity, restrict droplet mobility, and influence network organization [6]. Gellan gum (GG) is an anionic polysaccharide with strong hydration, thickening, and network-forming potential [7], making it a suitable material for modulating the physical and rheological properties of SPI-based emulsion gels. Because SPI and GG contribute differently to emulsifying behavior and bulk-phase structuring, their relative proportion may serve as an important formulation variable for controlling the overall properties of SPI–GG-based emulsion gels [8].

Maillard reaction-mediated conjugation is commonly used to prepare protein–polysaccharide conjugates by covalently linking amino groups of proteins with reducing carbonyl groups of carbohydrates [9]. The resulting conjugates can exhibit changes in protein dispersibility, surface hydrophobicity, charge characteristics, conformational properties, and emulsifying behavior [10]. For SPI–GG systems, the relative proportions of protein and polysaccharide are particularly important because composition can influence glycation-related characteristics and the physicochemical properties of the resulting conjugates. Importantly, previous studies indicate that the functional properties of protein–polysaccharide conjugates do not necessarily vary proportionally with polysaccharide content or degree of glycation [11].

Previous studies have shown that protein–polysaccharide Maillard conjugates can modify solubility, emulsifying properties, physical stability, and the delivery of lipophilic compounds [12]. SPI–GG conjugates have also been reported to exhibit composition- and glycation-dependent functionality [13]. However, limited information is available on whether variations in the SPI–GG ratio produce consistent changes across emulsion characteristics and bulk rheological properties within the same conjugate-stabilized emulsion-gel system, and how these composition-dependent responses relate to different food applications. This aspect is relevant because different food applications may place different demands on the material. Extrusion-based 3D printing requires shear-induced flow followed by sufficient structural recovery and post-deposition strength [14], whereas the delivery of lipophilic compounds is influenced by droplet organization, matrix stability, and digestion-related accessibility [15]. Evaluating these applications using the same formulation series can therefore clarify whether changes in SPI–GG composition produce similar or application-dependent functional responses.

Accordingly, this study investigated the effects of the SPI-to-GG mass ratio on the formation and physicochemical characteristics of Maillard-treated SPI–GG conjugates, as well as on the emulsion characteristics and bulk rheological properties of the resulting conjugate-stabilized emulsion gels (CEGs). Their functional performance was further evaluated through extrusion-based 3D printing and the encapsulation and *in vitro* bioaccessibility of β-carotene as a model lipophilic compound. The objective was to establish composition–property–function relationships in SPI–GG conjugate-stabilized emulsion gels, and to assess whether different SPI–GG ratios produce distinct functional responses depending on the intended application.

## Materials and Methods

### Materials

Soy protein isolate (SPI, protein content ≥ 90%) and low-acyl gellan gum (GG) were purchased from ES food (Republic of Korea). Sunflower oil was purchased from Herbnoori (Republic of Korea). β-Carotene and fluorescein isothiocyanate (FITC) were purchased from Sigma-Aldrich (USA), and Nile Red was obtained from Tokyo Chemical Industry (TCI, Japan). All other chemical reagents were of analytical grade, and distilled water was used throughout the experiments.

### Preparation of SPI–GG Conjugates

SPI and GG were dispersed in distilled water at SPI-to-GG mass ratios of 2:1, 1:1, 1:1.5, and 1:2 and stirred at room temperature for 2 h. Each dispersion was adjusted to pH 7.0 using 1 mol/L HCl, followed by hydration at 4°C for 24 h. The fully hydrated dispersion was then heated to 90°C for 60 min to induce conjugation. After heating, the samples were rapidly cooled to 25°C to terminate the reaction and subsequently stored at 4°C until further characterization and CEG preparation. The resulting SPI–GG conjugates were labeled S2G1, S1G1, S1G1.5, and S1G2, according to their respective SPI-to-GG mass ratios. A dispersion containing SPI alone was prepared under the same conditions and used as the control.

### Characterization of SPI–GG Conjugates

**Browning intensity and Glycation degree (GD).** The browning intensity (BI) of the SPI–GG conjugates was determined according to the method of [16]. The conjugates were freeze-dried at -85°C for 48 h, ground into a fine powder, and dispersed distilled water at a concentration of 5 mg/mL. Absorbance was measured at 294 nm and 420 nm using a UV-Vis spectrophotometer (SpectraMax ID3, Molecular Devices, USA).

The degree of glycation between SPI and GG was determined using an Ortho-phthaldialdehyde (OPA) assay according to [17] .The OPA reagent was prepared by dissolving 40 mg of OPA dissolved in 1 mL of methanol, followed by the addition of 100 μL of β-mercaptoethanol, 2.5 mL of 20% (w/w) sodium dodecyl sulfate, and 25 mL of 100 mM sodium borate buffer (pH 9.5). The final volume was adjusted to 50mL with deionized water. Prior to analysis, each SPI–GG conjugate dispersion was diluted with deionized water to an SPI-equivalent concentration of 2 mg/mL, calculated based on the nominal SPI content of each formulation. Subsequently, 200 μL of each diluted sample, corresponding to 0.4 mg of SPI, was mixed with 4 mL of OPA reagent. An unglycated SPI solution prepared at the same SPI concentration was used as the reference, while 200 μL of deionized water was used as the reagent blank.

The absorbance was measured at 340 nm using a UV-Vis spectrophotometer (SpectraMax ID3, Molecular Devices). The free amino group content was quantified using an L-lysine standard curve ranging from 10 to 500 μM (R^2^=0.99). The degree of glycation was calculated using the following equation:

$$\text{DG}(\%)=\left(1-\frac{A}{B}\right)\times 100$$

where *A* and *B* represent the free amino group content of the glycated SPI–GG conjugated and unglycated SPI reference, respectively.

**FTIR spectra.** The SPI–GG conjugates were subjected to freeze-drying at -85°C for 48 h prior to analysis. FTIR spectra were determined using a Cary 630 FTIR Spectrometer equipped with a diamond ATR accessory (Agilent Technologies, USA). Measurements were conducted in the wavenumber range of 4000 to 650 cm^−1^ with a resolution of 4 cm^−1^.

**Solubility and Surface Hydrophobicity (H_0_).** SPI and SPI–GG conjugates were diluted to a protein concentration of 2 mg/mL. The samples were then centrifuged at 10,000 rpm for 15 min at 4°C, and 1 mL of the supernatant was mixed with 4 mL of Biuret reagent. The resulting solutions were kept at room temperature for 20 min. The absorbance was measured at 540 nm using a UV–Vis spectrophotometer (SpectraMax ID3, Molecular Devices). The protein content was determined based on a bovine serum albumin (BSA) standard curve ranging from 0 to 10 mg/mL (*R^2^*=0.99). Solubility was then calculated with the following formula:

$$\text{Solubility}(\%)=\frac{P_{s}(\text{mg}/\text{ml})}{P_{i}(\text{mg}/\text{ml})}\times 100$$

where *P_s_* represents the protein concentration of supernatant, and *P_i_* represents the protein concentration of initial sample solution.

Surface hydrophobicity (H_0_) was measured using the fluorescent probe 8-anilinonaphthalene-1-sulfonic acid (ANS). SPI–GG conjugates were prepared at protein concentrations ranging from 0.05 to 1 mg/mL. And each solution was mixed with 20 μL of 8 mM ANS in 10 mM sodium phosphate buffer. The mixtures were incubated in the dark for 15 min. Fluorescence intensity was measured using a Spectro fluorophotometer (SpectraMax ID3, Molecular Devices) at an excitation wavelength of 390 nm and an emission wavelength of 470 nm. Surface hydrophobicity (H_0_) was determined as the slope of the linear regression curve between relative fluorescence intensity and protein concentration.

### Preparation of SPI–GG Conjugate-Stabilized Emulsion Gels (CEGs)

Conjugate-stabilized Emulsion gels (CEGs) were prepared using SPI–GG conjugates at a final concentration of 3.15% (w/v) and a sunflower oil phase fraction of 30% (w/w). The aqueous and oil phases were homogenized at 11,000 rpm for 3 min to form the conjugate-stabilized emulsions. Based on the SPI:GG mass ratios of 2:1, 1:1, 1:1.5, and 1:2, the resulting samples were designated as CEG-S2G1, CEG-S1G1, CEG-S1G1.5, and CEG-S1G2, respectively. The emulsions were subsequently stored at 4°C to induce gel formation.

### Characterization of SPI–GG Conjugate-Stabilized Emulsion Gels (CEGs)

**Droplet size and zeta potential.** The oil droplet distribution was determined using a laser diffraction analysis (Shimadzu SALD-2300, Japan). The CEGs were diluted at a of 1:100 ratio (w/v) with deionized water to obtain an appropriate particle concentration and minimize multiple scattering. The diluted samples were vortexed at 2,000 rpm for 1 min and subsequently introduced into the water-filled circulation cell of the instrument. The refractive index and adsorption coefficient were set to 1.5 and 0.1, respectively. Droplet size was expressed as the mean diameter, and all measurements were performed in triplicate.

The ζ-potential of the oil droplets was determined from their electrophoretic mobility using a ζ-potential analyzer (ELSZ-2000, Otsuka Electronics, Japan). The CEGs were diluted at a ratio of 1:100 (w/v) with deionized water and vortexed at 2,000 rpm for 1 min. The diluted samples were then transferred into disposable ζ-potential cells and analyzed in triplicate.

**Confocal laser scanning microscopy.** The microstructure of the CEGs was visualized using a confocal laser scanning microscopy (LSM 880 Airyscan, Carl Zeiss AG, Germany) equipped with a 40× oil-immersion objective. During CEG preparation, the oil and aqueous phases were stained with Nile Red at 0.1% (w/v) and fluorescein isothiocyanate (FITC) at 0.05% (w/v), respectively. Nile red and FITC were excited at 561 and 488 nm, and fluorescence emissions were detected at 595 and 525 nm, respectively. Images were acquired at a resolution of 1024 × 1024 pixels and processed using Zen software (Carl Zeiss AG).

**Storage stability.** Freshly prepared CEGs were transferred into glass vials and stored at 4°C for 30 days. Their visual appearance was recorded on 0, 7, 14, and 30 days to assess storage stability.

**Emulsifying properties.** The emulsifying activity index (EAI) and emulsion stability index (ESI) were determined based on the turbidity of diluted CEG samples. An aliquot (0.1 mL) of each freshly prepared CEGs was diluted to final volume of 10 mL with 0.1% (w/v) sodium dodecyl sulfate solution. The absorbance at 500 nm was measured immediately after dilution and after 10 min using a UV-Vis spectrophotometer (SpectraMax ID3, Molecular Devices). The EAI and ESI were calculated as follows:

$$\text{EAI}({\text{m}}^{2}/\text{g})=\frac{2\times 2,303\times A_{0}\times D}{c\times \phi \times L\times 10^{4}}$$

$$\text{ESI}(\text{min})=\frac{A_{0}}{A_{0}-A_{10}}\times 10$$

where A_0_ and A_10_ represent the absorbance measured at 0 min and 10 min, respectively; *D* is the dilution factor; *C* is the initial protein concentration; *Ø* is the oil volume fraction; and *L* is the optical length of the cuvette.

### Evaluation of Conjugate-Stabilized Emulsion Gels (CEGs) for Extrusion-Based 3D Printing

**Rheological characterization.** The rheological properties of the Conjugate-Stabilized Emulsion gels (CEGs) were measured using a rheometer (MCR 102, Anton Paar, Austria) equipped with a 25-mm parallel plate at a gap of 1.5 mm. Silicone oil was applied around the exposed edge of each sample to minimize moisture evaporation, and the data were analyzed using RheoCompass software (version 1.22, Anton Paar).

A strain sweep from 0.1% to 100% was performed at 1 Hz to determine the linear viscoelastic region (LVR). The critical strain (γc) was defined as the strain at which the storage modulus (G′) decreased by 5% from its plateau value within the LVR [18]. The apparent yield stress was determined by an oscillatory stress sweep from 1 to 1500 Pa at 1 Hz and defined as the crossover point of the storage modulus (G′) and loss modulus (G″). Flow behavior was evaluated by rotational shear-viscosity measurements conducted in flow mode over a shear rate range of 0.01 to 100 s^−1^, and the shear-thinning behavior was analyzed using the Power-law equation.

$$\eta =k{\left(\dot{\gamma }\right)}^{n-1}$$

where η is the apparent viscosity (Pa), $\dot{\gamma }$ is the shear rate (s^−1^), and K is consistency coefficient (Pa·s^n^) parameter and *n* is the flow behavior index.

Structural recovery was evaluated using a three-interval thixotropy test (3ITT). A low shear rate of 0.1 s^−1^ was first applied for 180 s to represent the resting state before extrusion, followed by a high shear rate of 100 s^−1^ for 120 s to simulate passage through the nozzle. The shear rate was then returned to 0.1 s^−1^ for 180 s to evaluate post-extrusion recovery. The recovery rate was calculated as the viscosity measured 30 s after the beginning of the third interval relative to the viscosity in the first interval.

**Extrusion-based 3D printing process.** A screw-based 3D printing system (CGBIO, Republic of Korea) was used to perform the 3D printability of the CEGs. Three-dimensional models were designed using CAD software (Inventor, USA), exported in stereolithography (STL) format, and converted into G-code using slicing software (Simplify3D, version 4.1.1, USA). The CEGs were extruded through an 18-gauge stainless-steel nozzle at a printing speed of 400 mm/min.

**Evaluation of printing accuracy.** Cuboid models with designed dimensions of 15 mm in width, 15 mm in depth, and 10 mm in height were used to evaluate the printing accuracy of the CEGs. The dimensions of the printed constructs were measured using a digital vernier caliper (Digital Caliper, Japan). The dimensional deviation from the original design was calculated for each direction using the following equation.

$$\text{Dimensional printing deviation}(\%)=\frac{\left(D_{p}-D_{d}\right)}{D_{d}}\times 100$$

where D_p_ was the printed dimension, and D_d_ was the designed dimension.

A positive value indicates that the printed dimension was larger than the designed dimension, whereas a negative value indicates that it was smaller. All printing experiments were performed in triplicate.

### Evaluation of Emulsion Gels as Lipophilic Bioactive Delivery Systems

**Preparation of β-carotene-loaded conjugate-stabilized emulsion gels.** For the preparation of β-carotene-loaded CEGs, β-carotene was first dissolved in sunflower oil at a concentration of 0.1% (w/v) to obtain a homogeneous oil phase. The β-carotene-containing oil phase was then mixed with the aqueous phase containing the SPI–GG conjugates and homogenized according to the procedure described in Section 2.4. Based on the SPI:GG mass ratios of 2:1, 1:1, 1:1.5, and 1:2, the resulting samples were designated as β-S2G1, β-S1G1, β-S1G1.5, and β-S1G2, respectively. After homogenization, the emulsions were stored at 4°C to promote gel formation.

**Encapsulation efficiency of β-carotene.** The encapsulation efficiency of β-carotene in the CEGs was determined according to the method of [19], with slight modifications. Each sample was mixed with an extraction solvent consisting of absolute ethanol and n-hexane at a volume ratio of 1:3 to extract the β-carotene retained within the CEG matrix. After shaking, the n-hexane phase containing β-carotene was collected. The extraction procedure was repeated three times to ensure sufficient recovery of β-carotene.

The absorbance of the extracted β-carotene was measured at 450 nm using a UV–Vis spectrophotometer (SpectraMax ID3, Molecular Devices). The amount of β-carotene extracted from the CEGs was quantified using a standard calibration curve (R^2^=0.99). The encapsulation efficiency (EE) was calculated using the following equation:

$$\text{EE}(\%)=\frac{\text{Encapsulated}\beta -\text{carotene}(\text{mg})}{\text{Total}\beta -\text{carotene}(\text{mg})}\times 100$$

Encapsulated β-carotene represents β-carotene in CEGs, and Total β-carotene input represents the total β-carotene added.

***In vitro* digestion and β-carotene bioaccessibility.** *In vitro* gastrointestinal digestion of the β-carotene-loaded CEGs was performed using the static INFOGEST protocol described by [20], with slight modifications. For the oral phase, each CEG sample (5 g) was mixed with simulated salivary fluid (SSF; pH 6.8) at a ratio of 1:1 (w/w) and incubated at 37°C for 5 min with continuous agitation at 200 rpm. For the gastric phase, the oral bolus was mixed with simulated gastric fluid (SGF; pH 2.5) at a ratio of 1:1 (w/w). Pepsin was added to achieve a final activity of 2000 U/mL, and the mixture was incubated at 37°C for 2 h with continuous agitation at 200 rpm. For the intestinal phase, the gastric digesta was mixed with simulated intestinal fluid (SIF; pH 7.0) at a ratio of 1:1 (w/w). Pancreatin was added to provide a final trypsin activity of 100 U/mL, together with lipase at a final activity of 2000 U/mL and bile salts at a final concentration of 10 mM. The mixture was then incubated at 37°C for 2 h with continuous agitation at 200 rpm. Following intestinal digestion, the digesta was centrifuged at 10,000 rpm for 30 min at 4°C. The intermediate aqueous layer was collected as the micellar fraction. β-Carotene in the micellar fraction was extracted using a mixture of n-hexane and ethanol at a volume ratio of 3:1. The absorbance of the extract was measured at 450 nm using a UV–Vis spectrophotometer (SpectraMax ID3, Molecular Devices). The *in vitro* bioaccessibility of β-carotene was calculated according to [21] using the following equation:

(N8) $$\text{Bioaccessibility}(\%)=\frac{C_{micelle}}{C_{Digesta}}\times 100$$

where *C_micelle_* is the concentration of β-carotene in micelle phase and *C_digesta_* is the concentration of β-carotene in the total digesta collected after the small intestine phase.

### Statistical Analysis

The results are presented as mean ± standard deviation (SD) values with each analysis conducted in triplicate (n=3). Statistical analysis was conducted using one-way ANOVA followed by Duncan’s multiple range test in SPSS 12.0 (SPSS, USA). Differences between means were evaluated based on least square means, and statistical significance was considered at *p* < 0.05. Graphical visualizations were generated using SigmaPlot version 12.5 (Systat Software, USA).

## Results and Discussion

### Formulation and Physicochemical Properties of SPI–GG Conjugates

**Degree of glycation and browning intensity.** In this study, SPI and gellan gum conjugates were prepared via Maillard reaction, and the apparent progression of the reaction was evaluated spectrophotometrically. Absorbance at 294 and 420 nm was used as an indirect indicator of the accumulation of intermediate products, including Schiff bases and Amadori products, and last-stage browning such as melanoidins, respectively [22]. Fig. 1A illustrates the absorbance at 294 nm and 420 nm for the different SPI–GG conjugate formulations. The absorbance at 294 nm decreased with increasing GG content, whereas absorbance at 420 nm showed the opposite trend. Because absorbance at 294 nm is geneally associated with colorless intermediate Maillard reaction products, while absorbance at 420 nm reflects the formation of brown products [23], this inverse pattern may indicate a relative shift from the accumulation of intermediate compounds toward the formation of later-stage browning products at higher GG proportions [24]. Subsequently, the degree of glycation (DG) was determined from the reduction in OPA-reactive free amino groups relative to unglycated SPI and was used as an indirect indicator of the extent of Maillard glycation [25]. As shown in Fig. 1B, the degree of glycation (DG) significantly increased as the proportion of GG increased (*p* < 0.05). The DG increased from approximately 16% in S2G1 to 21% in S1G1 and 26% in S1G1.5, reaching approximately 50% in S1G2. This trend indicates that a greater proportion of GG was associated with the consumption of free amino groups in SPI.

These results may be attributed to a change in the relative availability of reactive groups as the SPI–GG ratio varied. Increasing the GG proportion may have increased the probability of contact between GG-derived reducing-ends and accessible amino groups of SPI, thereby promoting amino group consumption within the tested formulation range. Similar behavior has been reported for SPI-maltodextrin conjugates, in which the highest degree of glycation was obtained at a protein-to-polysaccharide ratio of 1:2 [26]. Consistent with this trend, the concurrent increases in DG and absorbance at 420 nm indicate that higher GG proportions were associated with greater amino group involvement and more pronounced late-stage Maillard browning. Meanwhile, the decrease in absorbance at 294 nm may reflect a lower relative accumulation of intermediate products at the endpoint of the reaction rather than reduced glycation [27]. Overall, these results indicate that SPI–GG composition influenced the glycation-related characteristics of the conjugate systems under the tested reacted conditions [9, 28].

**Fourier transform infrared (FTIR) spectroscopy.** FTIR spectroscopy was used to characterize molecular interactions and structural changes in SPI–GG conjugates prepared at different ratios (Fig. 1C). The characteristic amide I and II bands were observed at 1630 and 1528 cm^−1^, respectively, whereas GG exhibited characteristic bands associated with hydroxyl, carboxylate, and C–O groups [29]. The spectra of the conjugates contained absorption features derived from both SPI and GG. Following conjugation, the amide I and II bands shifted to1612 and 1511 cm^−1^, respectively, and band broadening was observed in the 3200−3400 cm^−1^ region [30]. These spectral changes suggest that varying SPI–GG ratio altered the local chemical environment and intermolecular interactions within the conjugate systems [31]. The increase in DG indicated greater consumption of free amino groups, whereas the FTIR spectral changes reflected alterations in the molecular environment of the SPI–GG systems. Together with the browning and DG results, these spectral changes provide complementary evidence of Maillard-induced conjugation between SPI–GG [32, 33].

**Solubility and Surface Hydrophobicity (H_0_).** To characterize composition-dependent amphiphilic properties relevant to emulsion formation, the solubility and surface hydrophobicity (H_0_) of the SPI–GG conjugates were evaluated (Fig. 1D). Solubility reflects aqueous dispersibility, whereas H_0_ indicates the accessibility of hydrophobic groups that may contribute to affinity for the oil-water interface [34]. The solubility of SPI was approximately 30%, whereas S2G1 exhibited a markedly higher solubility of approximately 97%. Among the SPI–GG formulations, solubility progressively decreased with increasing GG proportion, reaching approximately 31% in S1G2, which was not significantly different from that of SPI (*p* > 0.05). In contrast, the surface hydrophobicity (H_0_) increases continuously with increasing GG proportion, and S1G2 exhibited the highest H_0_ value. These results demonstrate an inverse relationship between aqueous dispersibility, as reflected by solubility, and hydrophobic-group accessibility across the SPI–GG composition range.

The initial increase in solubility at lower GG proportion may be associaated with the hydrophilic contribution of GG chains and reduced protein-protein interactions. However, solubility progressively decreased as the GG proportion increased, whereas surface hydrophobicity (H_0_) showed the opposite trend [35]. This inverse relationship indicates that the SPI–GG ratio altered the balance between aqueous dispersibility and hydrophobic-group accessibility in the conjugate formulations. Increased surface hydrophobicity may enhance hydrophobic interactions among protein molecules, which could contribute to reduced aqueous solubility [36]. Consistently, previous work on SPI-gellan gum conjugates reported that formulations with lower glycation exhibited higher solubility and emulsifying properties but lower surface hydrophobicity than those with medium or high glycation levels [37]. These findings suggest that the relative SPI–GG composition modulates physicochemical characteristics relevant to emulsion formation, particularly the balance between aqueous dispersibility and hydrophobic-group accessibility. Therefore, solubility and H_0_ were subsequently considered in relation to the emulsifying behavior of the corresponding SPI–GG conjugate-stabilized systems.

### Emulsion Characteristics and Physical Stability of SPI–GG Conjugate Stabilized Emulsion Gels (CEGs)

To examine how the composition-dependent physicochemical characteristics of SPI–GG conjugate were associated with the properties of the resulting emulsion gels, droplet characteristics, emulsifying properties, and physical stability were evaluated across the SPI:GG ratios.

**Droplet characteristics and Microstructure.** To determine whether the composition-dependent properties of SPI–GG conjugates were reflected in the droplet organization of the resulting conjugate-stabilized emulsion gels (CEGs), droplet size, ζ-potential, and CLSM observations were analyzed together. As shown in Fig. 2A and B, all SPI–GG conjugate-stabilized emulsion gels exhibited smaller mean droplet diameters and more negative ζ-potential values than the SPI-stabilized emulsion gel. Similar reductions in droplet size and increases in ζ-potential magnitude have been reported in emulsions stabilized by protein–gellan gum conjugates and other protein–anionic polysaccharide conjugates [38, 39].

As the GG proportion increased from CEG-S2G1 to CEG-S1G1, the mean droplet diameter decreased, while the ζ-potential shifted to more negative values. CLSM images also showed visually finer and more evenly distributed droplets in CEG-S1G1. Among the tested formulations, CEG-S1G1 exhibited the smallest mean droplet size, the most negative ζ-potential, and a relatively uniform droplet distribution. These characteristics are generally associated with greater electrostatic repulsion and decreased tendency for droplet aggregation [37].

However, further increases in the GG proportion beyond CEG-S1G1 resulted in a gradual increase in droplet size and a shift in ζ-potential toward less negative values, accompanied by larger and more heterogenous droplets in the CLSM images. This reversal may be related to changes in both conjugate composition and aqueous dispersibility. Because the total conjugate concentration was maintained constant, increasing the GG proportion simultaneously reduced the relative SPI fraction in the formulation. In protein-stabilized emulsions, sufficient availability and transport of surface-active components during homogenization are important for stabilizing newly generated droplets, whereas limited availability can promote recoalescence and larger droplet formation [40, 41]. The marked decrease in solubility observed at higher GG proportions may have further reduced the availability and mobility of the conjugates in the aqueous phase. Previous studies have shown that extensive glycation and increased molecular size of protein–polysaccharide conjugates can reduce solubility and hinder their diffusion and rearrangement during emulsion formation [42, 43]. These factors may explain why the decrease in droplet size observed up to S1G1 was not maintained at higher GG proportions. Overall, the results indicate a non-monotonic effect of the SPI–GG ratio on droplet characteristics rather than a response proportional to GG content.

**Emulsifying properties and physical stability.** The emulsifying properties of the CEGs stabilized by SPI–GG conjugates were evaluated to determine how conjugate composition affected emulsifying performance (Fig. 3A). All CEGs exhibited higher EAI and ESI values than native SPI, with S1G1 showing the highest values of 18.32 ± 0.07 m^2^/g and 240.98 ± 9.02 min, respectively. Both indices increased from S2G1 to S1G1 but declined with further increases in the GG proportion, indicating a non-linear dependence on the SPI:GG ratio. Notably, the highest EAI and ESI values did not coincide with the maximum solubility or surface hydrophobicity (H_0_), which were observed for S2G1 and S1G2, respectively. This lack of correspondence indicates that emulsifying performance of the conjugates could not be attributed to either physicochemical property alone, but rather reflected their combined influence on aqueous dispersibility and hydrophobic-group accessibility [10]. Consistent with this interpretation, S1G1 also exhibited the smallest droplet size, the most negative ζ-potential, and a relatively uniform droplet distribution, as described in section 3.2.1. In contrast, the decline in EAI and ESI at higher GG proportions coincided with reduced solubility and less uniform droplet characteristics. Because increasing the GG proportion also reduced the relative SPI fraction at a constant total conjugate concentration, these changes may have limited the availability and mobility of surface-active components during emulsion formation [40]. Overall, the results show that emulsifying performance depended on the relative SPI–GG composition rather than increasing proportionally with GG content.

To examine whether the higher EAI and ESI values of the conjugates were accompanied by enhanced macroscopic stability of the resulting CEGs, their visual appearance was monitored during storage (Fig. 3B). The SPI-stabilized CEG exhibited progressive aqueous-phase separation and lost its self-supporting structure, whereas all conjugate-stabilized CEGs remained visually homogeneous and retained their structure upon inversion for up to 30 days. Similar suppression of creaming and oil release has been reported for concentrated emulsions stabilized by protein–polysaccharide Maillard conjugates [39]. The enhanced storage stability of the conjugate-stabilized CEGs was consistent with their smaller droplet sizes and retained macroscopic structure, which may have reduced droplet migration and phase separation during storage [44].

### Rheological Properties and 3D Printing Performance of Conjugate-Stabilized Emulsion Gels (CEGs)

**Shear-thinning behavior of inks during the extrusion stage.** The effects of the SPI–GG ratio on the characteristics of conjugate-stabilized emulsion gels were investigated in the preceding sections. To further evaluate their potential as structuring materials for food applications, the gels were assessed for extrusion-based 3D printing. Rheological properties corresponding to the extrusion, self-supporting, and structural recovery stages of the printing process were analyzed [45]. SPI was excluded from the subsequent rheological analysis because it failed to form a gel structure (Fig. 3B) and therefore did not possess the structural characteristics required for use as a printing ink.

During the extrusion stage, inks should exhibit shear-thinning behavior to ensure continuous and smooth flow through the nozzle without clogging [46]. As shown in Fig. 4, all CEG samples exhibited typical non-Newtonian shear-thinning behavior, with viscosity decreasing as the shear rate increased. This behavior may be attributed to the progressive alignment of oil droplets and the temporary breakdown of the gel network under increasing shear [47]. The shear-thinning behavior was further confirmed by fitting the viscosity data to the Power Law model. As summarized in Table 1, all samples exhibited flow behavior index (n) values below 1, confirming their shear-thinning characteristics [48]. Overall, all SPI–GG-based CEGs exhibited the fundamental shear-thinning behavior required for extrusion-based 3D printing. In addition, the consistency index (K) generally increased with increasing GG proportion, likely due to the enhanced viscosity imparted by the polysaccharide [49]. Such differences in consistency may influence the ease of extrusion and flow behavior during the printing process.

**Thixotropic behavior of inks during the recovery stage.** Following extrusion through the nozzle, the ink should rapidly recover its structure after the removal of shear to maintain the deposited shape and support subsequent layer formation [50]. Therefore, the structural recovery behavior of the conjugate-stabilized emulsion gel was evaluated using a three-step shear test. As shown in Fig. 5A, all CEGs exhibited a pronounced decrease in viscosity under high shear, followed by rapid viscosity recovery when the shear rate was reduced, reaching a relatively stable state within 30 s. Furthermore, all samples exhibited recovery rates above 70% (Fig. 5B), demonstrating favorable thixotropic behavior. These results indicate that SPI–GG-based conjugate-stabilized emulsion gel were capable of recovering their structure after extrusion, which may contribute to maintaining the integrity of deposited layers during 3D printing [51].

Among the formulations, CEG-S1G1 exhibited the highest recovery rate, suggesting the greatest ability to rapidly reorganize its internal structure following the removal of shear [52]. Beyond the CEG-S1G1 ratio, the recovery rate decreased as the proportion of GG increased. This trend may be associated with the intrinsic thickening and gel-forming properties of GG. At higher GG proportions, the increased consistency of the formulations may have limited the rate of structural rearrangement after shear, resulting in lower recovery within the measurement period. These findings suggest that the recovery behavior of the CEGs is governed by the balance between SPI–GG interactions and the extent of GG-dominated network formation [53].

**Viscoelastic behavior of inks during the self-supporting stage.** After structural recovery following extrusion, the deposited ink should possess sufficient mechanical strength and viscoelasticity to maintain its shape under its own weight and support subsequent layers [54]. As shown in Fig. 6A, all conjugated-stabilized emulsion gels exhibited G′ values consistently higher than G″ over the tested strain range, indicating predominantly solid-like behavior. Consistently, tan δ values remained below 1 for all samples (Table 2), confirming the dominance of elastic behavior [55].

The complex shear modulus (G*), which reflects the overall rigidity of a viscoelastic material, increased progressively with GG proportion, from 585.00 Pa for CEG-S2G1 to 2669.47 Pa for CEG-S1G2, indicating enhanced structural rigidity across the tested formulations. This increase may be associated with the greater contribution of GG to the continuous-phase network as its proportion increased [56]. However, G* decreased in CEG-S1G2 did not coincide with higher critical strain or yield stress, indicating that increased rigidity alone did not result in a corresponding improvement in all rheological properties related to self-supporting behavior. These combined effects may have contributed to the reduction in G*. The critical strain, defined as the strain at which G′ decreased by 5% from its plateau value [18], increased from 2.43% for CEG-S2G1 to 3.01% for CEG-S1G1 and 3.88% for CEG-S1G1.5, followed by a slight decrease to 3.60% for CEG-S1G2. Because the critical strain represents the upper limit of the linear viscoelastic region, a higher value indicates that the material can tolerate greater deformation before the onset of structural disruption [57]. Accordingly, CEG-S1G1.5 showed the highest critical strain among the tested formulations, whereas the slightly lower value of CEG-S1G2 indicates that its greater rigidity did not correspond to a greater tolerance to deformation before initiation from the LVR.

Yield stress, which reflects the resistance of the deposited ink to deformation during the self-supporting stage, showed a different composition-dependent pattern [58]. CEG-S1G1 and CEG-S1G1.5 exhibited relatively high yield stress values, whereas a marked decrease was observed for CEG-S1G2 (Fig. 6B). These results indicate that increasing the GG proportion progressively increased structural rigidity but did not result in a corresponding improvement in all rheological properties related to self-supporting behavior. Rather, CEG-S1G1.5 showed a more balanced combination of rigidity, resistance to the onset of nonlinear deformation, and yield resistance. Thus, the effect of the SPI–GG ratio on self-supporting behavior was composition-dependent rather than proportional to GG content.

**3D printing performance of conjugate-stabilized Emulsion gels (CEGs).** Based on the rheological characteristics described above, the practical printability of the CEGs was evaluated using extrusion-based 3D printing. As shown in Fig. 7A, all SPI–GG-based CEGs could be extruded through the nozzle, but their ability to retain the designed three-dimensional structure varied depending on the SPI–GG ratio.

The designed dimensions of the printed structures were 15 × 15 × 10 mm, and the actual dimensions are summarized in Table 3. CEG-S2G1 sample showed pronounced lateral spreading and loss of height, reaching 32.35 ± 4.62 mm in width and 31.98 ± 4.78 mm in depth, while its height decreased to 7.42 ± 0.97 mm (Fig. 7B). In contrast, CEG-S1G1, CEG-S1G1.5, and CEG-S1G2 maintained dimensions closer to the designed structure. Among these formulations, CEG-S1G1.5 showed the width and depth numerically closest to the target dimensions 15.96 ± 0.36 and 15.87 ± 0.30 mm, respectively), whereas CEG-S1G2 showed the closest height (9.99 ± 0.66 mm). The relatively small width and depth deviations observed for CEG-S1G1.5 are consistent with its relatively high G* and yield stress, suggesting that its bulk rheological characteristics contributed to post-deposition shape retention [59].

Notably, the formulation with the highest structural rigidity did not exhibit the smallest dimensional deviations. Although CEG-S1G2 showed the highest G*, its critical strain and yield stress were lower than those of CEG-S1G1.5. This indicates that post-deposition shape retention was not determined by rigidity alone but was associated with the combined resistance to small-deformation stiffness, strain-induced structural breakdown, and yielding. Moreover, CEG-S1G1, which exhibited the smallest droplets and the most negative ζ-potential, did not show the lowest dimensional deviations. Thus, the droplet-level characteristics associated with emulsion formation did not directly correspond to 3D-printing performance. Overall, the results indicate that post-deposition shape retention was not determined by rigidity alone but was associated with the combined effects of small-deformation stiffness, resistance to strain-induced structural breakdown, and yield resistance.

### β-Carotene Encapsulation and *In Vitro* Digestion Behavior

**Encapsulation efficiency.** As a separate application from extrusion-based 3D printing, the potential of CEGS for the encapsulation and delivery of lipophilic bioactive compounds was evaluated using β-carotene as a model compound. The effects of the SPI–GG ratio on β-carotene encapsulation efficiency were first investigated, followed by an assessment of β-carotene bioaccessibility using an *in vitro* digestion model.

As shown in Fig. 8A, the SPI-stabilized sample exhibited a relatively low encapsulation efficiency (EE) of 18.83 ± 4.38%, whereas all SPI–GG-conjugate based emulsion gels showed EE values exceeding 80%. Among the SPI–GG formulations, β-S1G1 exhibited the highest EE of 90.94 ± 0.55%, while β-S1G1.5 and β-S1G2 also maintained relatively high EE values. Among the SPI–GG formulations, β-S1G1 exhibited the highest EE of 90.94 ± 0.55%, while β-S1G1.5 and β-S1G2 also maintained relatively high EE values. Overall, β-carotene encapsulation varied depending on the SPI:GG composition, with β-S1G1 showing the highest encapsulation efficiency under the tested conditions [60].

***In vitro* bioaccessibility.** The *in vitro* bioaccessibility of β-carotene was further evaluated to determine the effect of the SPI–GG ratio on its incorporation into the micellar phase during digestion. As shown in Fig. 8B, the bioaccessibility of β-carotene in the β-SPI, β-S2G1, β-S1G1, β-S1G1.5, and β-S1G2 samples was 38%, 47%, 68%, 33%, and 32%, respectively, with β-S1G1 exhibiting the highest value. The higher bioaccessibility of β-S1G1 may be associated with the smaller and more uniformly distributed oil droplets observed for the corresponding CEG-S1G1 base formulation. The resulting larger oil–water interfacial area may facilitate lipase access to the droplet surface, promoting lipid digestion and the subsequent incorporation of β-carotene into mixed micelles [61]. In contrast, bioaccessibility decreased in β-S1G1.5 and β-S1G2 despite their relatively high encapsulation efficiencies. The increased contribution of GG to continuous-phase viscosity and gel structuring at higher GG proportions may restrict enzyme accessibility and mass transfer during digestion, thereby limiting lipid digestion and β-carotene micellarization [62]. These results indicate that high encapsulation efficiency does not necessarily result in high bioaccessibility. The composition-dependent differences in bioaccessibility may reflect both droplet-related characteristics affecting lipid digestion and matrix effects on enzyme accessibility and mass transfer. Thus, the SPI:GG ratio influenced β-carotene encapsulation and digestive bioaccessibility differently.

## Conclusion

In this study, Maillard conjugates were prepared at different the SPI–GG ratios and used as stabilizers to form conjugate-stabilized emulsion gels (CEGs) to investigate how variations in SPI–GG composition affect their physicochemical, emulsion, rheological, and functional properties. Browning intensity, degree of glycation, and FTIR analysis provided complementary evidence supporting conjugation between SPI and GG. The SPI–GG ratio produced distinct, non-linear changes in droplet characteristics, emulsifying properties, and bulk rheological behavior. the most negative ζ-potential, relatively uniform droplet distribution, and the highest EAI and ESI values. These characteristics coincided with higher β-carotene encapsulation efficiency and *in vitro* bioaccessibility, indicating that β-S1G1 was advantageous for lipophilic bioactive delivery. In contrast, CEG-S1G1.5 combined relatively high rigidity, the highest critical strain, high yield resistance, and low dimensional deviations during 3D printing, demonstrating a different composition requirement for structural performance. Further increasing the GG proportion to CEG-S1G2 did not result in proportional improvements across these properties. Overall, the results demonstrate that the functionality of SPI–GG conjugate-stabilized emulsion gels depend on composition-dependent relationships among physicochemical characteristics, droplet organization, and bulk rheological properties, and that the formulation providing the most suitable performance varies with the intended application. These findings provide a basis for composition-driven design of SPI–GG conjugate-stabilized emulsion gels for applications including 3D printing and lipophilic bioactive delivery.

## Figures and Tables

**Fig. 1:**
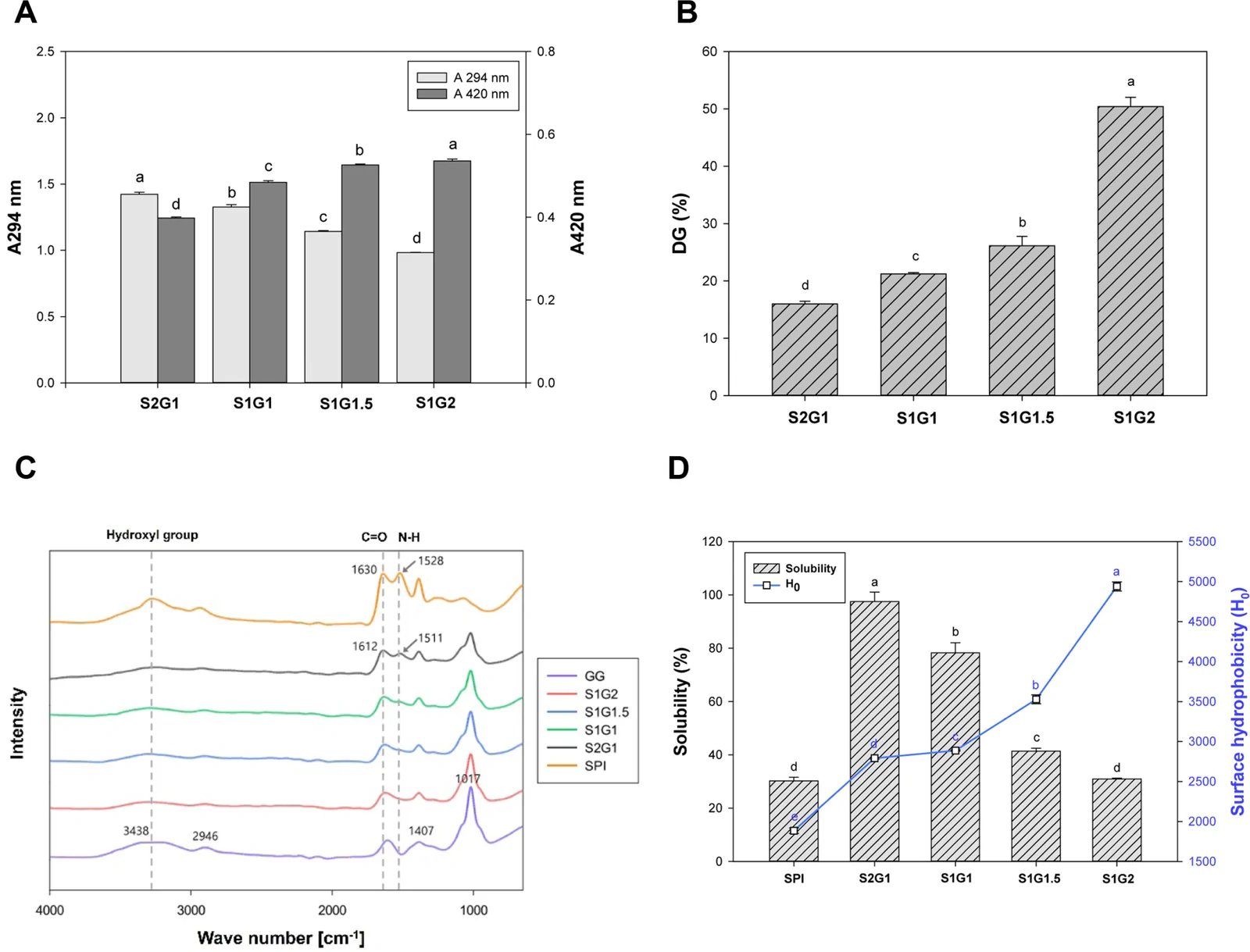
Physicochemical characterization of SPI–GG conjugates prepared at different SPI:GG ratios. (**A**) Absorbance at 294 and 420 nm, representing intermediate Maillard reaction products and browning intensity, respectively. (**B**) Degree of glycation (DG). (**C**) Fourier-transform infrared spectra of SPI, GG, and SPI–GG conjugates. (**D**) Solubility and surface hydrophobicity (H_0_). Different lowercase letters indicate significant differences among samples (*p* < 0.05).

**Fig. 2:**
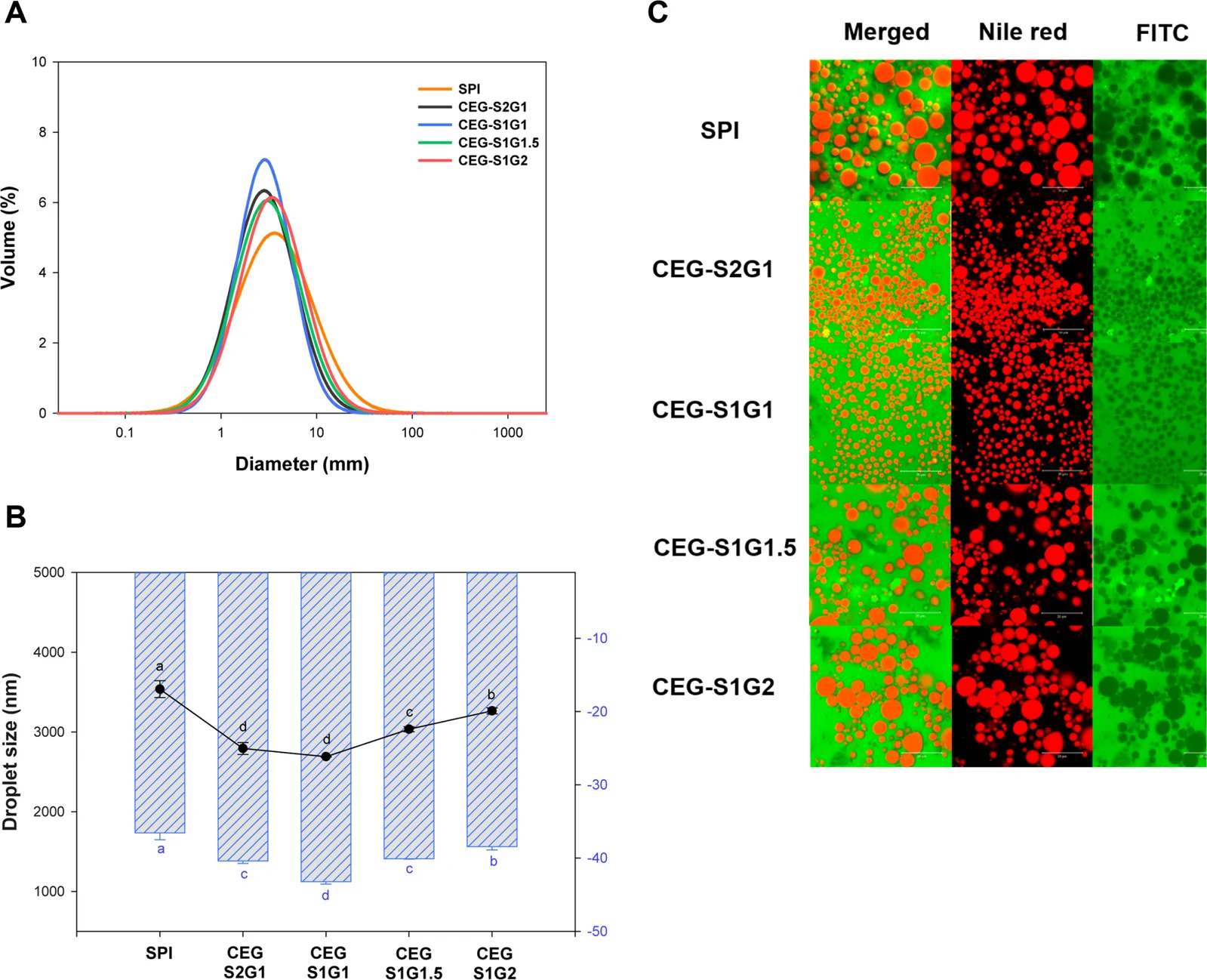
Droplet characteristics and microstructure of SPI–GG conjugate-stabilized emulsion gels. (**A**) Droplet size distribution. (**B**) Mean droplet size and zeta potential. (**C**) Confocal laser scanning microscopy images of the emulsions stabilized by SPI and SPI–GG conjugates. Nile red stained the oil phase, while FITC visualized the aqueous protein–polysaccharide phase. Different lowercase letters indicate significant differences among samples (*p* < 0.05). Scale bars = 20 μm.

**Fig. 3:**
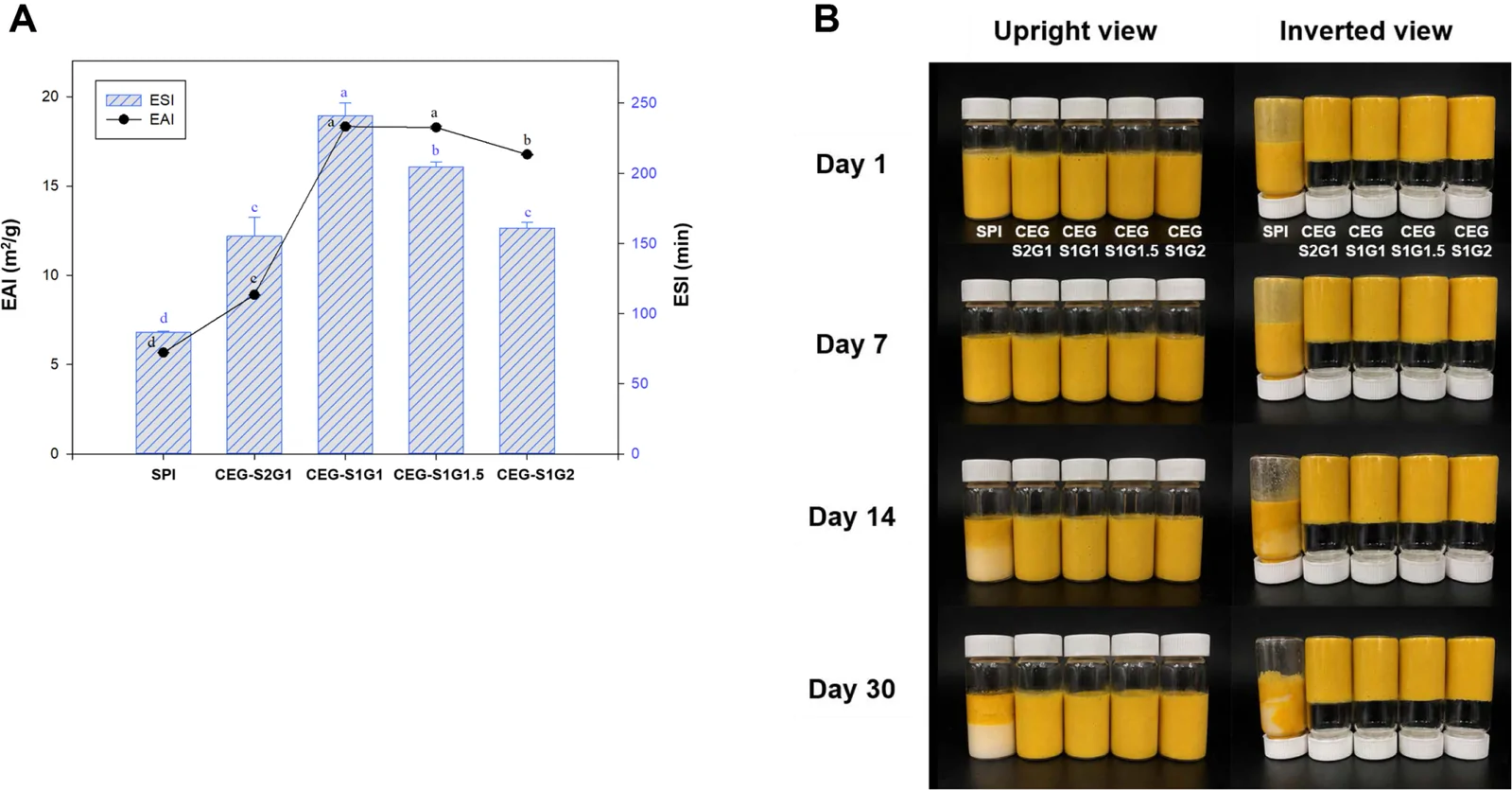
Emulsifying properties and storage stability of SPI–GG conjugate-stabilized emulsion gels. (**A**) Emulsifying activity index (EAI) and emulsion stability index (ESI) of SPI and SPI–GG conjugates. (**B**) Upright and inverted appearances of the resulting CEGs during storage for 1, 7, 14, and 30 days at 4°C. Different lowercase letters indicate significant differences among samples (*p* < 0.05).

**Fig. 4:**
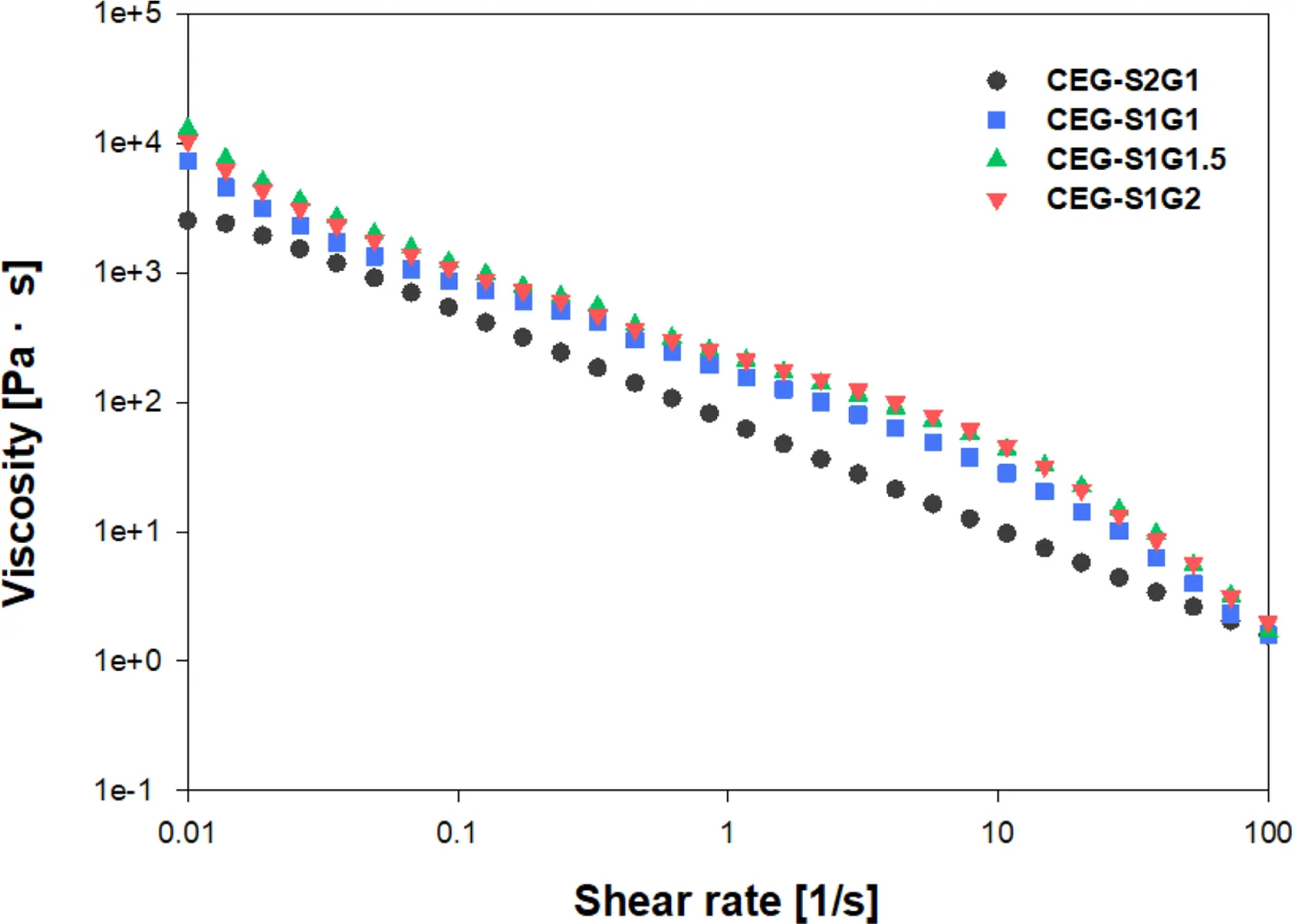
Flow behavior test of emulsion gels stabilized by SPI-GG conjugates with different SPI-GG mass ratios.

**Fig. 5:**
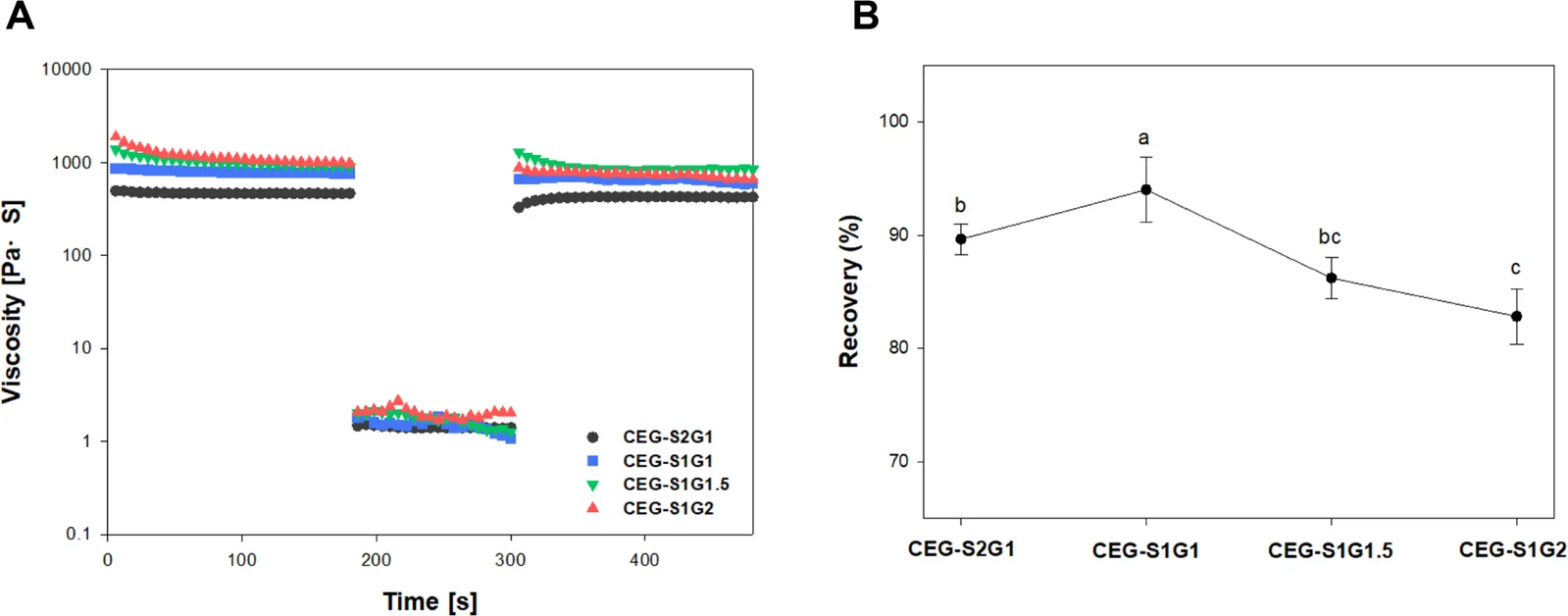
(A) 3ITT test and (B) recovery rate of emulsion gels stabilized by SPI-GG conjugates with different SPI-GG mass ratios.

**Fig. 6:**
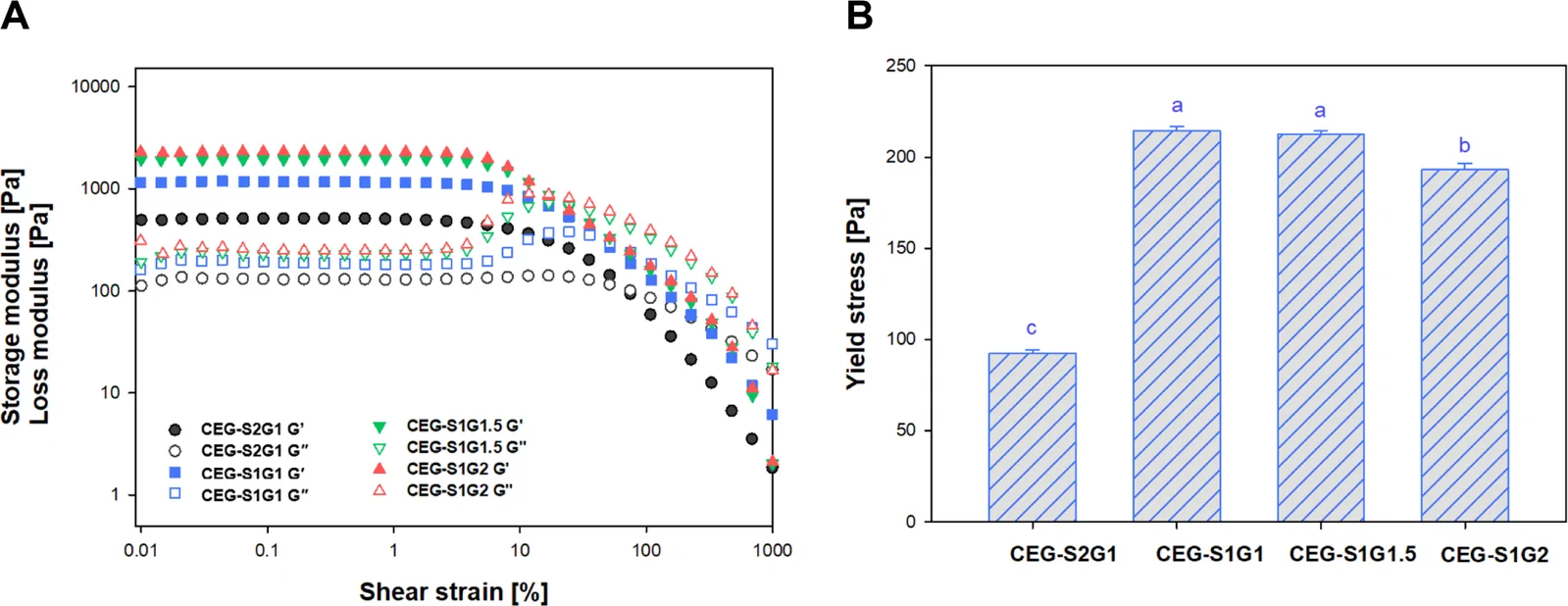
(A) Strain sweep test and (B) yield stress of emulsion gels stabilized by SPI-GG conjugates with different SPI-GG mass ratios.

**Fig. 7:**
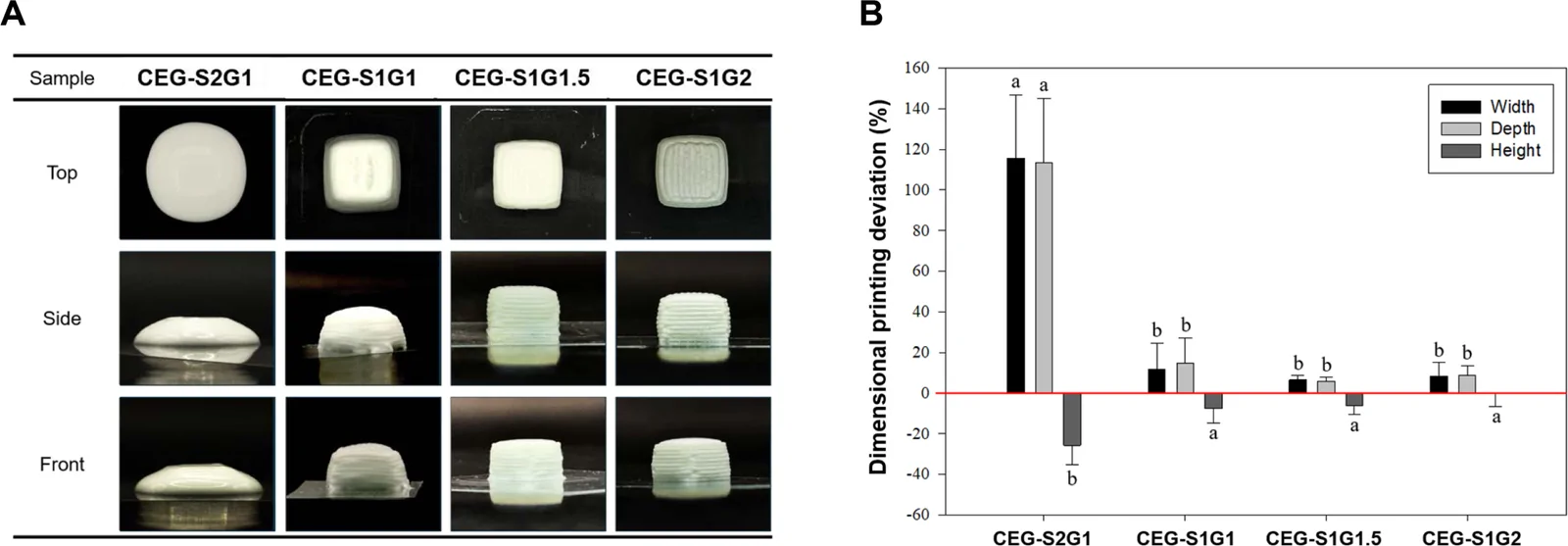
(A) 3D printed and (B) dimensional printing deviation of 3D printed cuboid structures of emulsion gels stabilized by SPI-GG conjugates.

**Fig. 8:**
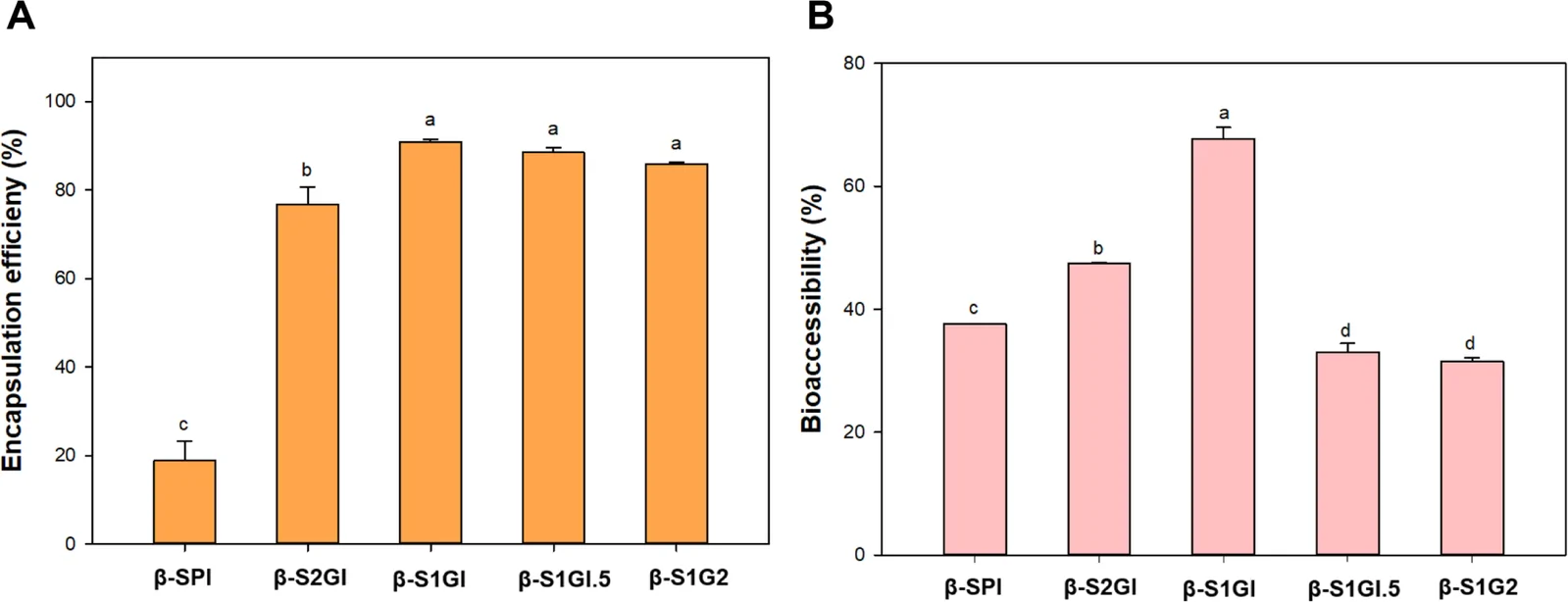
(A) Encapsulation efficiency and (B) bioaccessibility of β-carotene in emulsion gels stabilized by SPI and SPI-GG conjugates.

**Table 1:** Power-law model parameters describing the steady-shear flow behavior of SPI–GG conjugate-stabilized gels.

Sample^a^	K (Pa·s^n^)	n	R^2^
CEG-S2G1	69.60 ± 2.31^c^	0.18 ± 0.01^b^	1.00 ± 0.00^a^
CEG-S1G1	147.24 ± 4.00^b^	0.18 ± 0.01^b^	0.98 ± 0.00^b^
CEG-S1G1.5	207.75 ± 2.23^a^	0.18 ± 0.01^b^	0.98 ± 0.00^bc^
CEG-S1G2	204.21 ± 3.47^a^	0.20 ± 0.00^a^	0.98 ± 0.00^c^

^a^All processes were conducted three times. Values are the mean ± SD (n = 3). Values with different letters differ significantly at *p* < 0.05 (ANOVA with posthoc Duncan test).

**Table 2:** Viscoelastic, yield, and critical strain properties of SPI–GG emulsion gels.

Sample	G* (Pa)	Tan δ	Yield stress (Pa)	Critical strain (%)
CEG-S2G1	585.99 ± 14.98^d^	0.22 ± 0.00^a^	92.24 ± 2.22^c^	2.43%
CEG-S1G1	1262.53 ± 64.60^c^	0.13 ± 0.00^a^	214.53 ± 2.34^a^	3.01%
CEG-S1G1.5	2261.77 ± 70.57^b^	0.09 ± 0.00^a^	212.63 ± 1.63^a^	3.88%
CEG-S1G2	2669.47 ± 93.38^a^	0.08 ± 0.00^a^	193.07 ± 3.65^b^	3.60%

^a^All processes were conducted three times. Values are the mean ± SD (n = 3). Values with different letters differ significantly at *p* < 0.05 (ANOVA with posthoc Duncan test).

**Table 3:** Actual dimension of 3D printed conjugate-stabilized emulsion gels.

Sample	Width (mm)	Depth (mm)	Height (mm)
CEG-S2G1	32.35 ± 4.62^a^	31.98 ± 4.78^a^	7.42 ± 0.97^b^
CEG-S1G1	16.74 ± 1.91^b^	17.22 ± 1.84^b^	9.25 ± 0.74^a^
CEG-S1G1.5	15.96 ± 0.36^b^	15.87 ± 0.30^b^	9.36 ± 0.42^a^
CEG-S1G2	16.25 ± 0.99^b^	16.31 ± 0.73^b^	9.99 ± 0.66^a^

^a^All processes were conducted three times. Values are the mean ± SD (n = 3). Values with different letters differ significantly at *p* < 0.05 (ANOVA with posthoc Duncan test).
